# News and Miscellany

**Published:** 1900-05

**Authors:** 


### News and Miscellany.

---

**The Chas. H. Phillips Chemical Co.** removed May 1 to 128 Pearl Street, Rooms 4 and 5, New York.

---

**Dr. G. D. Lain** has purchased the property of Dr. E. H. Shelburne at Sanger and removed from Bolivar to that place.

---

**Dr. W. L. Davidson,** removed from Alleyton to Wharton; Dr. W. N. Lowe, from Sneed to Energy, Texas; Dr. J. A. Winfrey, from Winneville, I. T., to Hico, Texas.

---

**In Dr. Bat Smith's** article on yellow fever in our April issue the dose of bismuth in black vomit, he writes us, should have been stated at ʒi to ʒiss instead of ʒiii, as printed.

---

**Dr. A. Nolte,** of the Louisiana State Board of Health, honored the Texas State Medical Association by attending its four days sessions at Waco recently. Dr. Nolte made hosts of friends who will be glad to see him again.

---

**Dr. Turner,** late assistant physician at the Southwest Texas Insane Asylum at San Antonio has been appointed superintendent of the State Lunatic Asylum at Terrell, vice Wilson, resigned. Dr. Turner's successor at San Antonio has not been appointed at this date.

---

**Hon. W. L. Prather,** president of the University of Texas, will by invitation deliver the "commencement address" at the

University of Pennsylvania on June 13 (prox). It is understood that the University of Pennsylvania will on that occasion confer on Col. Prather the degree of LL. D. The commencement address last year was delivered by President McKinley and in 1898 by the Chinese ambassador.

---

**Dr. C. M. Rosser,** of Dallas, has accepted the position of medical referee for the Security Mutual Life Insurance Co. of New York. He will remain in Dallas.

---

**A Mare's Nest.**—The JOURNAL has received in the last few days letters from several of its advertisers, manufacturers of popular and valuable products, stating that they have authority for saying that at the approaching meeting of the American Medical Association at Atlantic City, "foreign chemical houses will attempt to elect their allies as chairman and members of the Section on Materia Medica with a view to discarding American products and endorsing only the foreign." We are asked to expose this "plot." Our correspondents add that "many good men are in it without realizing the nature of the underhanded scheme." The May number has been delayed long beyond its usual time of publication, hence we are enabled to get in the above; June would be too late. There is no danger of the American Medical Association "endorsing" any foreign proprietary article. We have room for no comments further than to add that no reputable physician would lend himself to any such "plot," and therefore we don't believe it.

